# Altered Respiratory Microbiomes, Plasma Metabolites, and Immune Responses in Influenza A Virus and Methicillin-Resistant Staphylococcus aureus Coinfection

**DOI:** 10.1128/spectrum.05247-22

**Published:** 2023-06-15

**Authors:** Qichao Chen, Manjiao Liu, Hao Guo, Kaiying Wang, Jiangfeng Liu, Yun Wang, Yanfeng Lin, Jinhui Li, Peihan Li, Lang Yang, Leili Jia, Juntao Yang, Peng Li, Hongbin Song

**Affiliations:** a Academy of Military Medical Sciences, Academy of Military Sciences, Beijing, China; b Center for Disease Control and Prevention of Chinese PLA, Beijing, China; c State Key Laboratory of Translational Medicine and Innovative Drug Development, Jiangsu Simcere Diagnostics Co., Ltd., Nanjing City, Jiangsu Province, China; d Nanjing Simcere Medical Laboratory Science Co., Ltd., Nanjing City, Jiangsu Province, China; e State Key Laboratory of Medical Molecular Biology, Department of Biochemistry and Molecular Biology, Institute of Basic Medical Sciences, Chinese Academy of Medical Sciences, School of Basic Medicine, Peking Union Medical College, Beijing, China; f School of Public Health, China Medical University, Shenyang City, Liaoning Province, China; University of Guelph College of Biological Science

**Keywords:** IAV-MRSA coinfection, respiratory microbiomes, plasma metabolites, host immunity, *Lactobacillus murinus*, adaptive immunity, bacterial-viral coinfection, innate immunity

## Abstract

Influenza A virus (IAV)–methicillin-resistant Staphylococcus aureus (MRSA) coinfection causes severe respiratory infections. The host microbiome plays an important role in respiratory tract infections. However, the relationships among the immune responses, metabolic characteristics, and respiratory microbial characteristics of IAV-MRSA coinfection have not been fully studied. We used specific-pathogen-free (SPF) C57BL/6N mice infected with IAV and MRSA to build a nonlethal model of IAV-MRSA coinfection and characterized the upper respiratory tract (URT) and lower respiratory tract (LRT) microbiomes at 4 and 13 days postinfection by full-length 16S rRNA gene sequencing. Immune response and plasma metabolism profile analyses were performed at 4 days postinfection by flow cytometry and liquid chromatography-tandem mass spectrometry (LC-MS/MS). The relationships among the LRT microbiota, the immune response, and the plasma metabolism profile were analyzed by Spearman’s correlation analysis. IAV-MRSA coinfection showed significant weight loss and lung injury and significantly increased loads of IAV and MRSA in bronchoalveolar lavage fluid (BALF). Microbiome data showed that coinfection significantly increased the relative abundances of Enterococcus faecalis, Enterobacter hormaechei, Citrobacter freundii, and Klebsiella pneumoniae and decreased the relative abundances of Lactobacillus reuteri and Lactobacillus murinus. The percentages of CD4^+^/CD8^+^ T cells and B cells in the spleen; the levels of interleukin-9 (IL-9), interferon gamma (IFN-γ), tumor necrosis factor alpha (TNF-α), IL-6, and IL-8 in the lung; and the level of mevalonolactone in plasma were increased in IAV-MRSA-coinfected mice. L. murinus was positively correlated with lung macrophages and natural killer (NK) cells, negatively correlated with spleen B cells and CD4^+^/CD8^+^ T cells, and correlated with multiple plasma metabolites. Future research is needed to clarify whether *L. murinus* mediates or alters the severity of IAV-MRSA coinfection.

**IMPORTANCE** The respiratory microbiome plays an important role in respiratory tract infections. In this study, we characterized the URT and LRT microbiota, the host immune response, and plasma metabolic profiles during IAV-MRSA coinfection and evaluated their correlations. We observed that IAV-MRSA coinfection induced severe lung injury and dysregulated host immunity and plasma metabolic profiles, as evidenced by the aggravation of lung pathological damage, the reduction of innate immune cells, the strong adaptation of the immune response, and the upregulation of mevalonolactone in plasma. *L. murinus* was strongly correlated with immune cells and plasma metabolites. Our findings contribute to a better understanding of the role of the host microbiome in respiratory tract infections and identified a key bacterial species, *L. murinus*, that may provide important references for the development of probiotic therapies.

## INTRODUCTION

Seasonal influenza is a global public health challenge. Bacterial superinfections are serious complications of influenza A virus (IAV) infection (1). Methicillin-resistant Staphylococcus aureus (MRSA), which is resistant to most antibiotics and transmitted in both the community and hospitals (2), often coinfects patients infected with IAV, causing severe lung injury and constituting an important global health concern (3). The host microbiome plays an important role in the pathogenesis of respiratory tract infections by resisting colonization by pathogens and promoting immune tolerance (4, 5). The depletion of the respiratory tract and gut microbiota by broad-spectrum antibiotic cocktails inhibited the synthesis of a proliferation-inducing ligand, resulting in secondary IgA deficiency and enhanced susceptibility to Pseudomonas aeruginosa infection in a murine pneumonia model and human intensive care unit (ICU) patients (6). In adults, the upper respiratory tract (URT) microbiome is stable under normal conditions but may be altered by infection, inflammation, or chronic respiratory diseases (7). IAV may disrupt the upper respiratory tract microbiome and increase the abundance of *Pseudomonadales* in ferrets (8). The pathogenesis of postinfluenza MRSA infections can be divided into IAV infection and IAV-MRSA coinfection stages. Most studies have focused on dysbiosis during the IAV infection stage; for example, in a murine model, IAV restructured the URT microbiota through the activation of host type III interferon production and subsequently increased susceptibility to MRSA infection (9). In addition, persistent disruption of the lower respiratory tract (LRT) microbiota was observed after IAV infection in mice and may predispose to bacterial invasion (10). However, current research has analyzed mainly the microbiota of the respiratory or intestinal tract in IAV-infected hosts and paid less attention to the correlation between the microbiota and the host immune response in IAV-MRSA coinfection. To elucidate the relationship among the immune response, plasma metabolites, and respiratory tract microbiota dysbiosis, we established a nonlethal IAV-MRSA coinfection mouse model and characterized the respiratory tract microbiome at 4 and 13 days postinfection by full-length 16S rRNA gene sequencing. We analyzed the host immune response and plasma metabolite profiles at 4 days postinfection by flow cytometry and liquid chromatography-tandem mass spectrometry (LC-MS/MS) and the correlations among the lower respiratory tract microbiota, immune cells, and plasma metabolites by ’Spearman’s correlation analysis.

## RESULTS

### IAV-MRSA coinfection induced acute severe lung injury.

**FIG 1:**
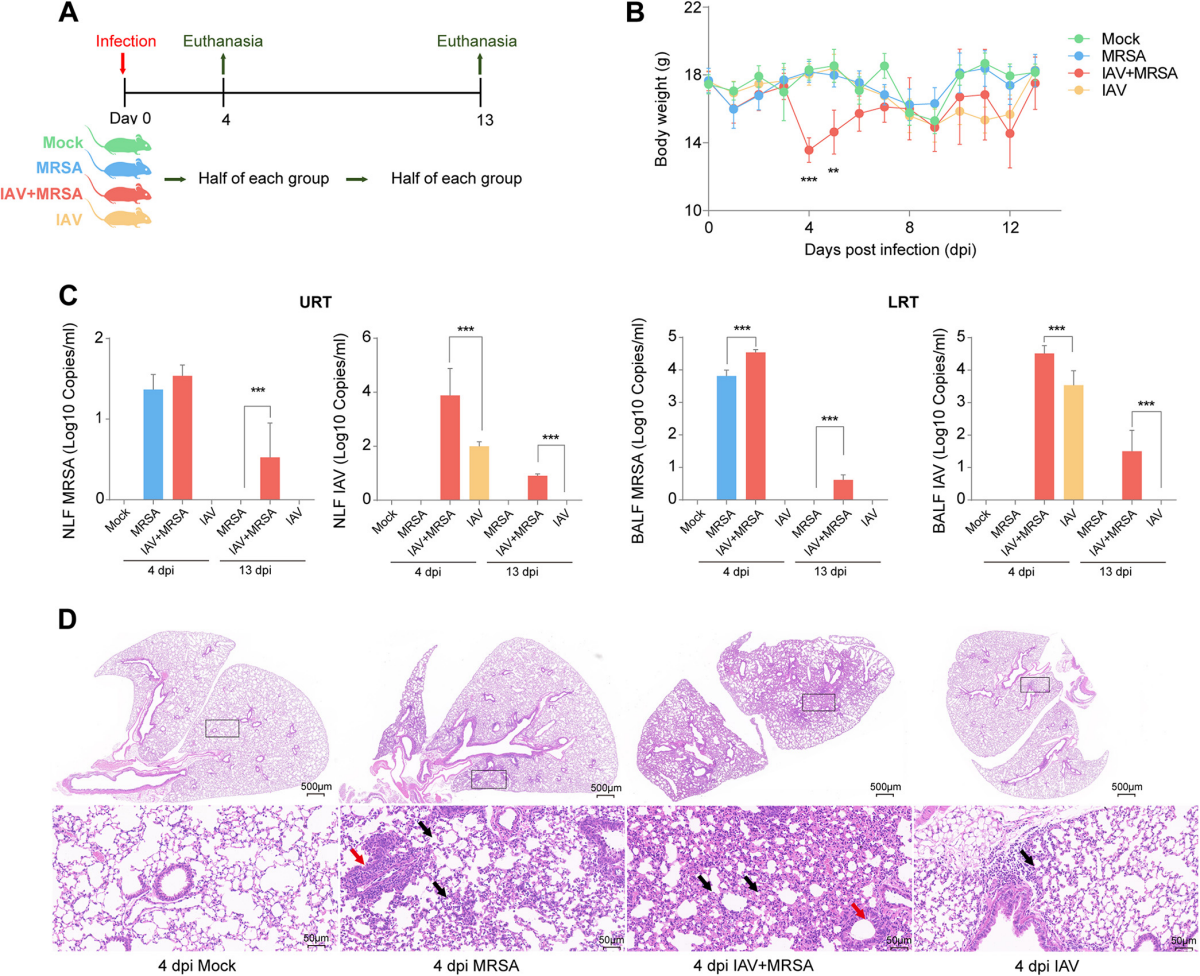
IAV-MRSA coinfection induced severe lung injury. (A) Challenge models of C57BL/6N mice infected with PBS (mock), methicillin-resistant Staphylococcus aureus (MRSA), influenza A virus (IAV), and IAV-MRSA. (B) Body weights of all groups. (C) Quantification of IAV and MRSA loads in nasopharyngeal lavage fluid (NLF) and bronchoalveolar lavage fluid (BALF) at 4 and 13 days postinfection (dpi) (*n* = 3 biological replicates for IAV and *n* = 3 biological replicates for MRSA per group). The mock group samples from 4 days after mock infection were set as the baseline samples. (D) Hematoxylin and eosin (H&E) staining of right lung tissues at 4 days postinfection (*n* = 3 biological replicates). Statistical analyses of pathogen loads were conducted by one-way ANOVA (**, *P < *0.01; ***, *P < *0.001).

The group coinfected with IAV and MRSA (IAV+MRSA group) on average weighed significantly less (*P < *0.05) than the mock group at 4 and 5 days postinfection but recovered at 13 days postinfection (Fig. 1B). Compared to the IAV group, the IAV loads in the bronchoalveolar lavage fluid (BALF) and nasopharyngeal lavage fluid (NLF) were significantly increased (*P < *0.05) in the IAV+MRSA group at 4 and 13 days postinfection. In the IAV+MRSA group, significantly increased loads of MRSA were observed (*P < *0.05) in BALF at 4 and 13 days postinfection and in NLF at 13 days postinfection (Fig. 1C). In the MRSA and IAV+MRSA groups, the alveolar lumen was infiltrated by lymphocytes, neutrophils, and macrophages (Fig. 1D, black arrows); moreover, inflammatory cells infiltrated around the blood vessels and formed a vascular cuff (red arrows). In the IAV group, the infiltration of lymphocytes and macrophages was observed in the lung tissue (Fig. 1D, black arrows). Compared to the mock group, the lung histological scores were significantly increased in the IAV+MRSA group (*P < *0.05), while the MRSA and IAV groups showed no significant difference. (Fig. 1D; see also Fig. S4 in the supplemental material).

### IAV-MRSA coinfection caused persistent changes in the respiratory microbiome.

**FIG 2:**
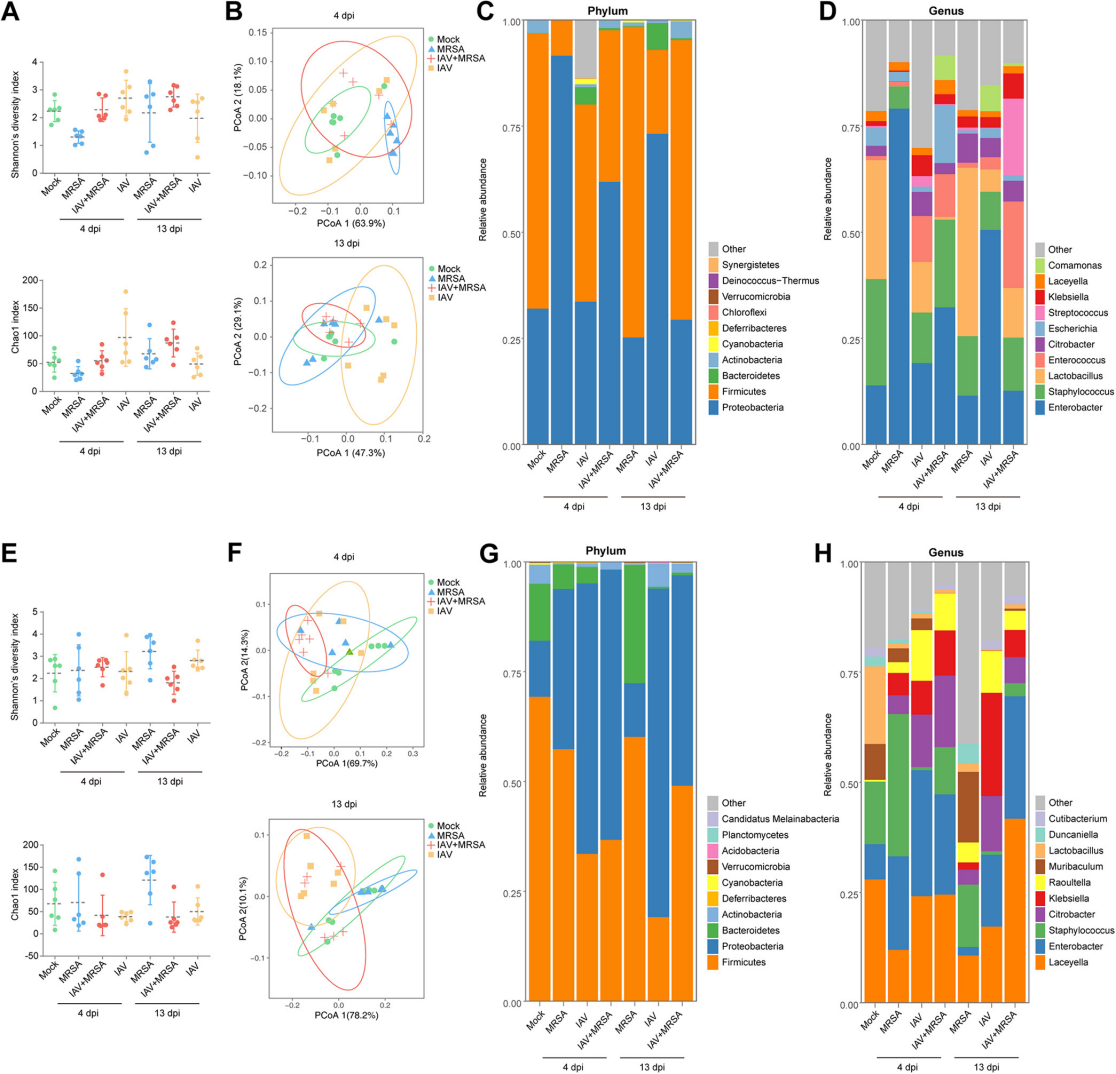
Characterization of the upper and lower respiratory tract microbiota in infected and mock-infected mice at 4 and 13 days postinfection. (A) Shannon diversity and Chao1 indices of the URT. (B) Principal-coordinate analysis of the URT microbiota. (C) Relative abundances of the top 10 members of the URT microbiota at the phylum level. (D) Relative abundances of the top 10 members of the URT microbiota at the genus level. (E) Shannon diversity and Chao1 indices of the LRT. (F) Principal-coordinate analysis of the LRT microbiota. (G) Relative abundances of the top 10 members of the LRT microbiota at the phylum level. (H) Relative abundances of the top 10 members of the LRT microbiota at the genus level (*n* = 6 biological replicates per group). The mock group samples from 4 days after mock infection were set as the baseline samples. Statistical analyses of the Chao1 and Shannon indices were conducted by using the Kruskal-Wallis test.

The abundance and diversity of the BALF and NLF microbiota showed no significant differences in the mono- or coinfection groups at either 4 or 13 days postinfection (Fig. 2A and E). The Bray-Curtis distance data from principal-coordinate analysis (PCoA) showed a significant separation (*P < *0.05) of the mock group from the IAV+MRSA group in the LRT, but no change was observed in the URT at 4 days postinfection. The separation of the IAV+MRSA group from the mock group was significantly increased (*P* < 0.05) in the URT at 13 days postinfection (Fig. 2B and F and Fig. S5). For all groups, *Firmicute*s and *Proteobacteria* dominated the LRT and URT microbiota. The IAV, MRSA, and IAV+MRSA groups showed a decrease in the relative abundance of *Firmicutes* but an increase in that of *Proteobacteria* in the LRT at 4 and 13 days postinfection. The IAV+MRSA group also showed a decrease in the relative abundance of *Firmicutes* but an increase in that of *Proteobacteria* at 4 days postinfection, whereas this disorder had been restored at 13 days postinfection in the URT. At the genus level, the relative abundance of *Lactobacillus* was decreased in both the LRT and URT in the IAV+MRSA group at 4 and 13 days postinfection. The relative abundances of *Enterococcus* and Enterobacter were increased in the URT, while those of *Citrobacter* and Klebsiella were increased in the LRT (Fig. 2C, D, G, and H).

**FIG 3:**
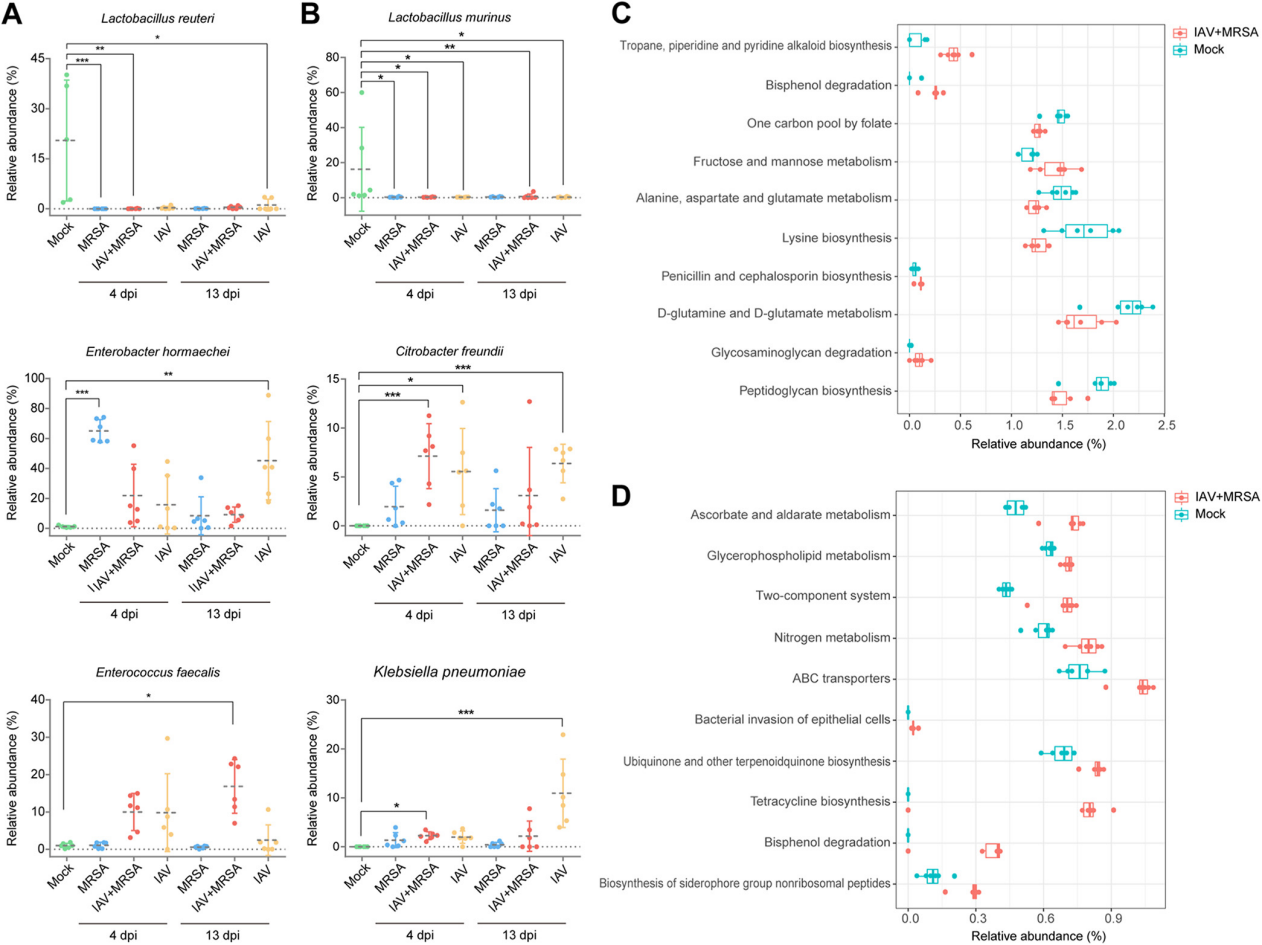
Altered relative abundances of members of the respiratory tract microbiota and corresponding metabolic changes during IAV-MRSA coinfection. (A) Relative abundances (percentages) of key bacteria at the species level in the URT microbiome. (B) Relative abundances (percentages) of key bacteria at the species level in the LRT microbiome. Statistics for relative abundance were analyzed by the Kruskal-Wallis test (*, *P < *0.05; **, *P < *0.01; ***, *P < *0.001). (C) KEGG metabolism pathway predictions for the URT microbiota at 4 days postinfection. (D) KEGG metabolism pathway predictions for the LRT microbiota at 4 days postinfection. All statistically significant changes were identified between the IAV+MRSA- and mock-infected groups. The top 10 corresponding pathways are represented in the bar plots (*n* = 6 biological replicates per group). The mock group samples from 4 days after mock infection were set as the baseline samples.

To further identify key members of the respiratory microbiome during IAV-MRSA coinfection, we analyzed the composition of the microbiota at the species level among all groups by the Kruskal-Wallis test. For the URT microbiota, compared with the mock group, the relative abundances of Enterococcus faecalis and Enterobacter hormaechei were increased slightly while that of Lactobacillus murinus was decreased significantly (*P < *0.05) in the IAV+MRSA group at 4 days postinfection. The relative abundance of E. faecalis was significantly increased (*P < *0.05) while the relative abundance of Lactobacillus reuteri was significantly decreased in the IAV+MRSA group compared with the mock group at 13 days postinfection. For the LRT microbiota, the relative abundances of Citrobacter freundii and Klebsiella pneumoniae were significantly increased (*P < *0.05) while that of *L. murinus* was decreased in the IAV+MRSA group at 4 days postinfection. The disorders of C. freundii, K. pneumoniae, and L. murinus in the IAV+MRSA group still had not been recovered at 13 days postinfection (Fig. 3A and B). In addition, compared with the IAV group at 4 days postinfection, the relative abundance of S. aureus was significantly increased (*P < *0.05) in the LRT while those of E. faecalis and E. hormaechei were significantly increased (*P < *0.05) in the URT in the IAV+MRSA group. Compared with the MRSA group, the relative abundance of *E. hormaechei* was significantly increased (*P < *0.05) in the LRT at 13 days postinfection while that of E. faecalis was significantly increased (*P < *0.05) in the URT at 4 and 13 days postinfection in the IAV+MRSA group (Table S2 and Fig. S6). Predicted KEGG metabolism pathways showed that the enrichments of d-glutamine and d-glutamate metabolism in the URT and ABC transporters in the LRT were significantly decreased and increased, respectively (*P < *0.05), at 4 days postinfection in the IAV+MRSA group compared to the mock group. In addition, compared to the IAV group, the enrichment of carbon fixation in photosynthetic organisms was significantly decreased (*P < *0.05) in the LRT while that of the pentose phosphate pathway was significantly increased (*P < *0.05) in the URT at 4 days postinfection in the IAV+MRSA group. Compared with the MRSA group, the enrichment of cysteine and methionine metabolism was significantly increased (*P < *0.05) in the LRT while that of C_5_-branched dibasic acid metabolism was significantly decreased (*P < *0.05) in the URT at 4 days postinfection in the IAV+MRSA group (Fig. S7 and S8).

### IAV-MRSA coinfection dysregulated the host immune response.

**FIG 4:**
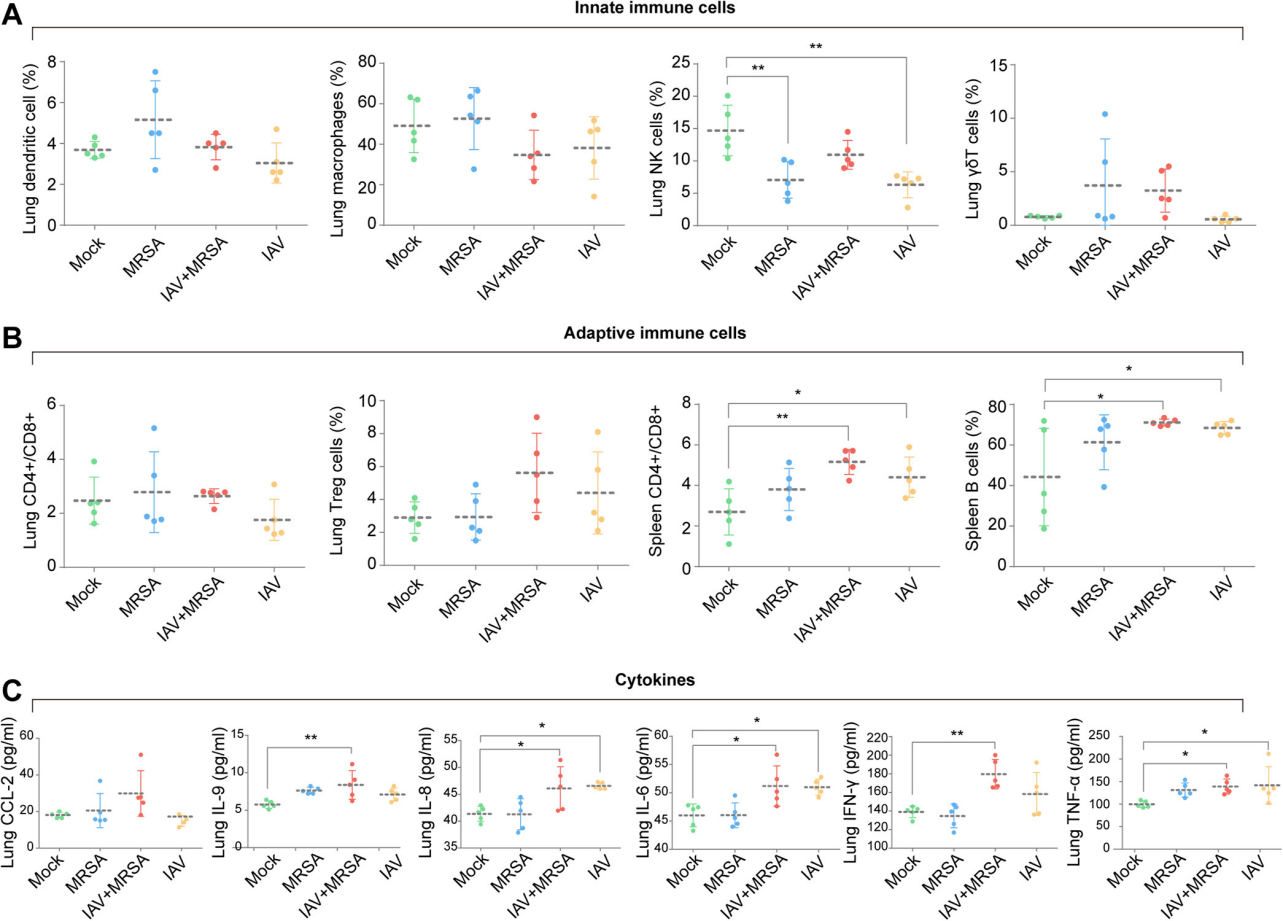
Immune cell and cytokine levels in the lung and spleen at 4 days postinfection. (A) Levels of innate immune cells (dendritic cells, macrophages, NK cells, and γδT cells) in the lung. (B) Levels of adaptive immune cells in the lung (CD4^+^/CD8^+^ T cells and Treg cells) and spleen (CD4^+^/CD8^+^ T cells and B cells). (C) Cytokine levels (CCL-2, IL-9, IL-6, IL-8, IFN-γ, and TNF-α) in the lung (*n* = 5 biological replicates per group). Statistics for immune cells and cytokines at 4 days postinfection were calculated by one-way ANOVA (*, *P < *0.05; **, *P < *0.01).

For lung innate immune cells, compared to the mock group, macrophage and natural killer (NK) cell levels were decreased in the IAV+MRSA group, while γδT cell levels were increased in the IAV+MRSA group, but the changes were not significant. For adaptive immune cells, both the IAV+MRSA and IAV groups showed slightly increased levels of lung regulatory T (Treg) cells and significant increases (*P < *0.05) in splenic CD4^+^/CD8^+^ and B cells (Fig. 4B). For lung cytokines, the IAV+MRSA group showed significantly increased levels of interleukin-9 (IL-9), interferon gamma (IFN-γ), tumor necrosis factor alpha (TNF-α), IL-6, and IL-8, while the IAV group showed significantly increased (*P < *0.05) levels of IL-6, IL-8, and TNF-α, compared to the mock group (Fig. 4C).

### IAV-MRSA coinfection modulates host plasma metabolic profiles.

**FIG 5:**
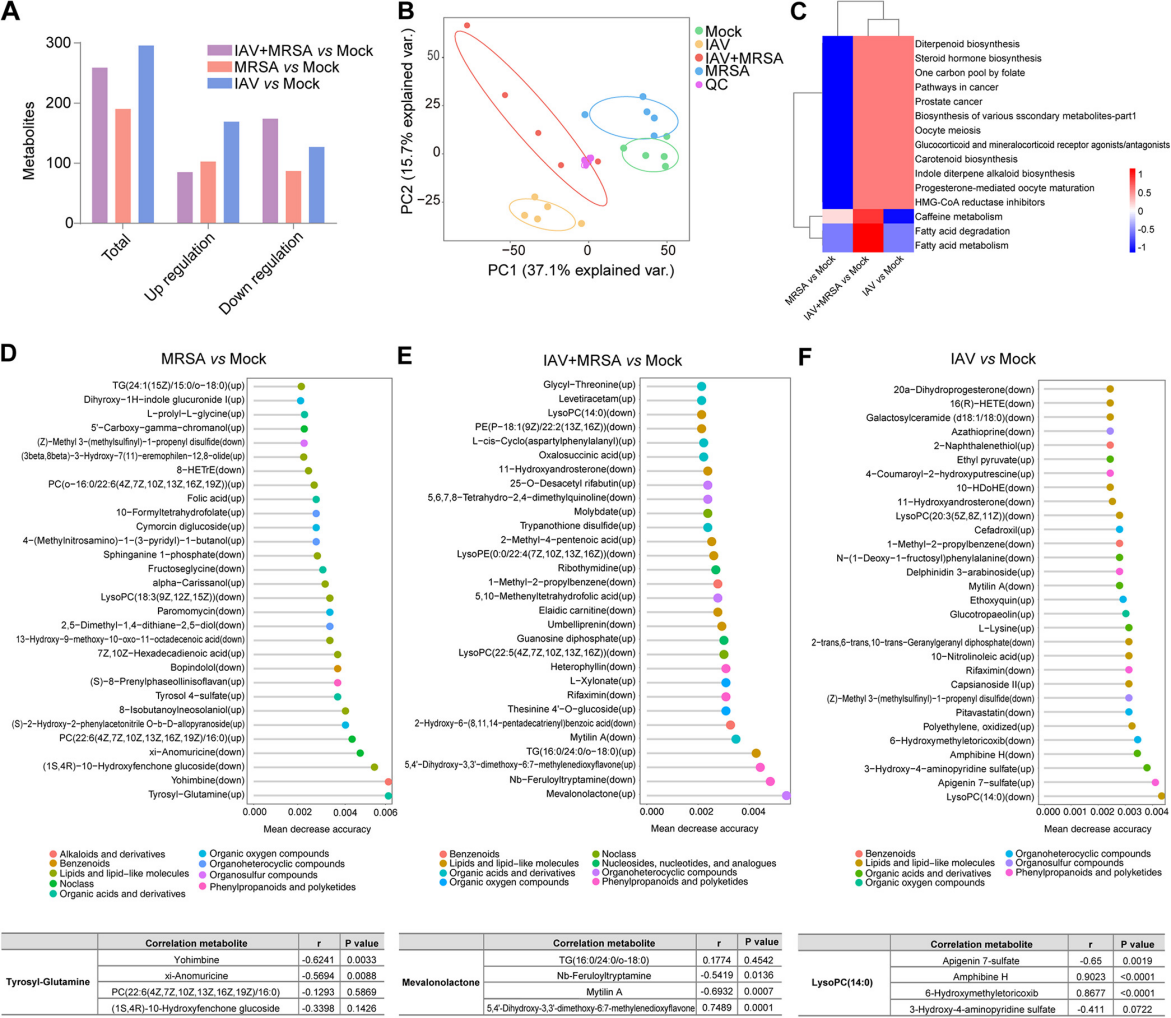
Plasma metabolic profiling at 4 days postinfection. (A) Numbers of significantly increased and decreased plasma metabolites (*P* value of <0.05; *q* value of <0.1) in all groups at 4 days postinfection by ANOVA contrasts. (B) Principal-coordinate analysis of plasma metabolites in all groups. (C) Increased and decreased enriched metabolic pathways in the IAV (influenza A virus), MRSA (methicillin-resistant Staphylococcus aureus), and IAV+MRSA groups compared to the mock-infected group. (D) The top 30 most distinguishing metabolites in the MRSA group compared to the mock-infected group by random-forest analysis and correlation analysis of tyrosyl-glutamine and the top 4 plasma metabolites. (E) The top 30 most distinguishing metabolites in the IAV+MRSA group compared to the mock-infected group by random-forest analysis and correlation analysis of mevalonolactone and the top 4 plasma metabolites. (F) The top 30 most distinguishing metabolites in the IAV-infected group compared to the mock-infected group by random-forest analysis and correlation analysis of lysophosphatidylcholine (lysoPC) and the top 4 plasma metabolites (*n* = 5 biological replicates per group). TG, Triacylglycerol; 8-HETrE, (8S,9Z,11E,14Z)-8-Hydroxyicosa-9,11,14-trienoate; 16(R)-HETE, (5Z,8Z,11Z,14Z,16R)-16-hydroxyicosa-5,8,11,14-tetraenoic acid; 10-HDoHE,(4Z,7Z,11Z,13Z,16Z,19Z)-10-Hydroxydocosa-4,7,11,13,16,19-hexaenoate; PE, phosphatidylethanolamine.

A total of 1,267 metabolites were identified in plasma. Compared with the mock group, the MRSA, IAV, and IAV+MRSA groups showed significant increases in 103, 169, and 85 metabolites and significant decreases in 87, 127, and 174 metabolites, respectively (Fig. 5A and Fig. S9). According to the characteristics of the metabolic profiles, PCoA showed a clear shift in the metabolites in the IAV and IAV+MRSA groups compared to the mock group, while the MRSA and mock groups showed similar metabolic profiles (Fig. 5B and Fig. S10). The IAV+MRSA and IAV groups showed similar enriched metabolic pathways, including diterpenoid biosynthesis, pathways in cancer, prostate cancer, 1-carbon pool by folate, and steroid hormone biosynthesis. In addition, the metabolites of the IAV+MRSA group showed significant enrichments (*P < *0.05) in fatty acid metabolic and degradation pathways (Fig. 5C). The top 15 metabolites were screened out to distinguish the other groups from the mock group, including elevated mevalonolactone levels in the IAV+MRSA group, decreased lysophosphatidylcholine (lysoPC) levels in the IAV group, and elevated tyrosyl-glutamine levels in the MRSA group (Fig. 5D to F).

### Host immune responses were associated with altered LRT microbial composition and plasma metabolites.

**FIG 6:**
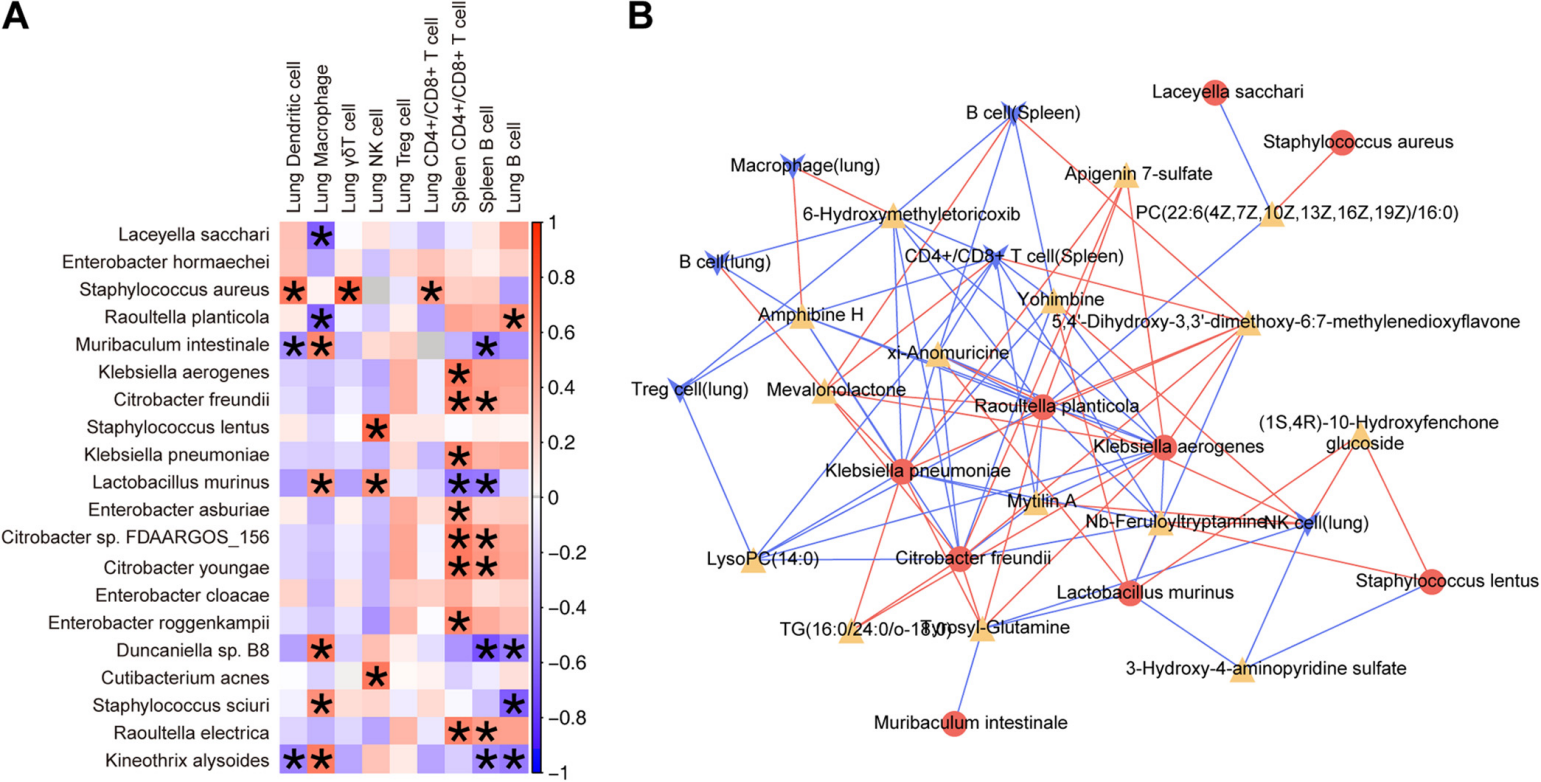
Analysis of the correlations among the lower respiratory tract (LRT) microbiota, immune cells, and plasma metabolites. (A) Correlations between host immune cells and the top 20 LRT bacterial taxa at the species level. Data with Spearman correlation coefficients of <0.5 are marked with *. (B) Negative and positive correlations of immune cells (blue arrows), the top 15 plasma metabolites (yellow triangles), and the top 10 bacterial species (red dots) were identified by Spearman analysis. Positive and negative correlations are indicated by red and blue lines, respectively. Only data with Spearman correlation coefficients of <0.5 are shown (*n* = 5 biological replicates per group).

Heat map analysis showed a strong correlation between the LRT microbiota and host immune cells. Pulmonary macrophages were positively correlated with Staphylococcus sciuri, *L. murinus*, Muribaculum intestinale, Kineothrix alysoides, and *Duncaniella* sp. strain B8 and negatively correlated with Raoultella planticola and Laceyella sacchari. Splenic CD4^+^/CD8^+^ lymphocytes were positively correlated with C. freundii, *Citrobacter* sp. strain FDAARGOS_156, Citrobacter youngae, Enterobacter asburiae, Enterobacter roggenkampii, Klebsiella aerogenes, K. pneumoniae, and Raoultella electrica and negatively correlated with *L. murinus* (Fig. 6A). *L. murinus* was significantly associated with both host innate and adaptive immunity, including positive correlations with lung macrophages and lung NK cells and negative correlations with splenic B cells and CD4^+^/CD8^+^ T cells. Interaction networks revealed that Raoultella planticola, C. freundii, K. aerogenes, and K. pneumoniae were increased in the IAV+MRSA group and were positively correlated with 5,4′-dihydroxy-3,3′-dimethoxy-6:7-methylenedioxyflavone, apigenin 7-sulfate, tyrosyl-glutamine, and mevalonolactone but negatively correlated with 6-hydroxymethyletoricoxib, amphibine H, lysoPC(14:0), mytilin A, nb-feruloyltryptamine, and xi-anomuricine. *L. murinus* was decreased in the IAV+MRSA group and was negatively correlated with 3-hydroxy-4-aminopyridine sulfate, 5,4′-dihydroxy-3,3′-dimethoxy-6:7-methylenedioxyflavone, and tyrosyl-glutamine but positively correlated with xi-anomuricine, yohimbine, nb-feruloyltryptamine, and (1*S*,4*R*)-10-hydroxyfenchone glucoside (Fig. 6B).

## DISCUSSION

Multiple studies have revealed that the respiratory tract microbiota plays a key role in the pathogenesis of IAV-bacterial coinfection (11). However, previous studies have characterized mainly the respiratory tract microbiota, not linking the microbiota to host immunity or metabolism in IAV-MRSA coinfection. Our study analyzed changes in the URT and LRT microbiota, host immune responses, and plasma metabolites and screened for key species through correlation analysis.

The IAV-MRSA-coinfected mice in our study showed significant weight loss compared with MRSA- or IAV-infected mice. Previous studies have reported severe weight loss in IAV-Streptococcus pneumoniae or IAV-Klebsiella oxytoca coinfections compared to monoinfections (12, 13). The weight loss caused by IAV-MRSA coinfection may be related to muscle catabolism and adipose tissue depletion due to the host’s immune response to pathogens (14). We observed that the MRSA and IAV loads in the LRT of coinfected mice were higher than those in MRSA- or IAV-infected mice. IAV has been demonstrated to impair IL-33 production and inhibit the immune system’s ability to clear MRSA (15). Moreover, an S. aureus factor (lipase 1) may enhance IAV replication via the positive modulation of virus budding (16). Synergistic interactions between MRSA and IAV may be responsible for severe lung injury and weight loss in coinfected mice. Microbiome analysis revealed that *Proteobacteria* and *Firmicutes* were the most dominant members in both the UTR and LRT, which showed an increase of *Proteobacteria* or a decrease of *Firmicutes* under IAV-MRSA infection. Disorders of *Firmicutes* and *Proteobacteria* were also observed previously in the LRT or gut of IAV-infected mice (17–19). Both Lactobacillus reuteri and *L. murinus* belong to the *Firmicutes* and were decreased in the URT and LRT of IAV-MRSA-infected mice. They were previously found to benefit the host immune system and could inhibit colonization by pathogenic microorganisms (20, 21). As opportunistic pathogens, E. faecalis, *E. hormaechei*, C. freundii, and K. pneumoniae belong to the *Proteobacteria* and were increased in the URT and LRT of IAV-MRSA-coinfected mice, which had been previously reported to cause severe infections (22–25). These results suggest that IAV-MRSA coinfection causes the depletion of the resident microbiota and elevated loads of opportunistic pathogens in the respiratory tract of mice, which may be important in the exacerbation of lung injury. Glutamine is an essential nutrient of macrophages, neutrophils, and lymphocytes and is required for the production of cytokines and the killing of pathogens (26). ABC transporter inhibition limits cytomegalovirus infection in infected cells (27, 28). Predicted function analysis of the microbiome showed that d-glutamine and d-glutamate were significantly reduced in the URT and that ABC transporters were significantly increased in the LTR the of IAV+MRSA group, indicating that the disordered respiratory microbiota plays an important role in host immunity.

Interestingly, we observed decreased levels of macrophages and NK cells and increased levels of γδT cells and Treg cells in the lungs of IAV-MRSA-infected mice. Pulmonary macrophages, as general housekeeping cells, play an important role in resistance to infection (29). A model of severe acute respiratory syndrome coronavirus 2 (SARS-CoV-2) and S. pneumoniae coinfection also showed alveolar macrophage depletion (30). Moreover, IAV accelerates macrophage apoptosis via its polymerase basic protein 1 frame 2 accessory protein (31). γδT cells are critically involved in immune surveillance and display multiple immunosuppressive mechanisms (32). Pandemic H1N1 infection induces γδT cell infiltration into lung tissue (33). Treg cells may limit immune-mediated pathology but may also reduce pathogen clearance (34). IAV-infected mice exhibited an increase in Treg cells in the lung tissue (35).

The splenic immune effector cell profiles of IAV-MRSA-coinfected mice were similar to those of IAV-monoinfected mice. All of the profiles showed increases in CD4^+^/CD8^+^ T cells and B cells. The abnormal ratio of CD4^+^/CD8^+^ T cells, as an indicator of immune regulation, suggests immune dysfunction. IAV infection increases virus-specific CD4^+^ T cells (36). In an IAV infection model, the humoral response consisted of innate-like B-1 B cells and virus-specific adaptive B cells (37). Cytokines are important determinants of disease severity. Our study shows that pulmonary IL-9, IFN-γ, TNF-α, IL-6, and IL-8 were significantly increased in IAV-MRSA-coinfected mice. IFN-γ, TNF-α, IL-6, and IL-8 are proinflammatory factors that are increased in other IAV-MRSA coinfection models (38). IL-9 is often considered to be positively associated with immunosuppression and IAV infection (39, 40). These results suggested that IAV-MRSA coinfection causes immune system suppression in the host, which may be a result of IAV infection. Mevalonolactone (upregulated) was the most discriminatory plasma metabolite in IAV-MRSA-coinfected mice. Mevalonolactone inhibits hydroxymethylglutaryl-coenzyme A reductase activity to modulate host mevalonic acid levels (41). The intermediate mevalonate is essential for inducing trained innate immunity (42). Moreover, prolonged treatment with mevalonolactone induces mitochondrial damage and inflammation in human glioblastoma U-87 MG cells (43). Elevated levels of mevalonolactone may be closely related to the suppression of intrinsic host immunity.

Through correlation analysis, we found that *L. murinus* was closely related to host immune cell levels and plasma metabolite levels. *L. murinus* belongs to the *Lactobacillus* genus, which can improve host IL-22 transcription by producing the aryl hydrocarbon receptor (AHR) ligand indole-3-aldehyde (44). IL-22 is known to be key to maintaining mucosal immunity. *L. murinus* can also increase IL-10 release through Toll-like receptor 2 (TLR2) and can alleviate murine intestinal ischemia/reperfusion (I/R) injury (45). In addition, the oral administration of *L. murinus* prevented late-onset sepsis in a murine model of neonatal intestinal dysbiosis caused by maternal antibiotic exposure (46). These results suggested that *L. murinus* may be a potential target for alleviating IAV-MRSA infection.

### Conclusions.

We characterized chronic changes in the URT and LRT microbiota during IAV-MRSA coinfection and observed that IAV-MRSA coinfection dysregulated host immunity and plasma metabolic profiles, as evidenced by the reduction of innate immune cells, the strong adaptive immune response, and the upregulation of mevalonolactone. Finally, we analyzed the correlation among host immune cells, the LRT microbiota, and plasma metabolites and found that *L. murinus* was directly and indirectly correlated with the immune response and may be a potential target to mitigate IAV-MRSA coinfection.

## MATERIALS AND METHODS

### Viruses and bacteria.

The influenza virus strain A/Puerto Rico/8/34 (H1N1) (PR8) was grown in embryonated chicken eggs, and its titer was determined by a 50% tissue culture infective dose (TCID_50_) assay in Madin-Darby canine kidney cells, as described previously (17, 47). The PR8 virus (10^−5.6^ TCID_50_/mL) solution contained 10^−4^ to 10^−1.6^ TCID_50_/mL for IAV-MRSA coinfection. MRSA strain BJFAN203 was grown in LB containing 50 μg/mL kanamycin. Cultures grown overnight were diluted 1:100 and grown for an additional 5 h to reach ~1 × 10^9^ CFU/mL. Bacteria were pelleted at 5,000 × *g* for 10 min and resuspended in 25 μL of phosphate-buffered saline (PBS) at an estimated final number of 8 × 10^7^ CFU for IAV-MRSA coinfection (48).

### Animals and sample collection.

Eighty specific-pathogen-free (SPF) C57BL/6N mice (female, 6 weeks of age) were purchased from Beijing Vital River Laboratory Animal Technology Co., Ltd. (Beijing, China), and raised under SPF/biosafety level (BSL2) conditions at the Technical Institute of Physics and Chemistry of the Chinese Academy of Science. After 1 week of adaptation, mice were randomly divided into four groups (*n *= 20 per group): PBS mock infection (mock), IAV-infected (IAV), MRSA-infected (MRSA), and IAV-MRSA-coinfected (IAV+MRSA) groups. For the IAV- or MRSA-infected group, anesthetized mice were infected intranasally with 25 μL of the A/Puerto Rico/8/34 (H1N1) strain (10^−1.6^ TCID_50_/mL) or MRSA strain BJFAN203 (8 × 10^7^ CFU), respectively. To establish a nonlethal model of IAV-MRSA coinfection, mice were infected with 25 μL of PR8 (10^−1.6^ TCID_50_/mL) and 0 days later with 8 × 10^7^ CFU of MRSA. Control animals were mock infected with 25 μL of sterile PBS. All mice had access to water and food during the experiment under a strict 12-h-light/dark cycle. Half of the mice in each group were euthanized at 4 and 13 days postinfection, respectively (Fig. 1A). Nasopharyngeal lavage fluid (NLF) and bronchoalveolar lavage fluid (BALF) at 4 and 13 days postinfection were collected as reported previously (38, 49). Plasma specimens were collected at 4 days postinfection and stored at −80°C for further experiments. The right lungs and spleens were collected at 4 days postinfection for flow cytometry, hematoxylin and eosin (H&E) staining, and enzyme-linked immunosorbent assays (ELISAs).

### Nucleic acid extraction and qPCR.

Total RNA was extracted using a QIAamp viral RNA minikit (Qiagen, Germany) according to the manufacturer’s instructions. Microbial DNA was extracted using a QIAamp ultraclean production pathogen minikit (Qiagen, Germany) according to the manufacturer’s instructions. Briefly, 400 μL of samples and 100 μL of tissue lysis buffer were homogenized in a pathogen lysis tube at 50 Hz for 10 min, and 400 μL of the supernatant was used for further DNA extraction steps. A control extraction with sterile PBS was performed for each kit. The MRSA bacterial load was measured using a methicillin-resistant Staphylococcus aureus probe quantitative PCR (qPCR) kit (Tiandz, China) according to the manufacturer’s instructions. The IAV titer was determined with a Luna universal probe one-step reverse transcription-qPCR (RT-qPCR) kit (New England BioLabs [NEB], USA) using forward primer 5′-GACCRATCCTGTCACCTCTGAC-3′, reverse primer 5′-GGGCATTYTGGACAAAKCGTCTACG-3′, and probe 5′-FAM (6-carboxyfluorescein)-TGCAGTCCTCGCTCACTGGGCACG-BHQ1 (black hole quencher 1)-3′ as recommended by the WHO (50). Copies of MRSA and IAV were determined using a standard curve.

### Full-length 16S rRNA gene sequencing.

DNAs extracted from NLF and BALF at 4 days postinfection and 14 days postinfection were used for full-length 16S amplification with primers 27F (5′-AGAGTTTGATCCTGGCTCAG-3′) and 1492R (5′-TACGACTTAACCCCAATCGC-3′). PCR was performed with Kapa HiFi HotStart ReadyMix (Kapa Biosystems, USA) and 0.5 mM forward and reverse primers. Cycling conditions were set at 95°C for 3 min; 30 cycles of 98°C for 20 s, 55°C for 30 s, and 72°C for 1 min; 72°C for 5 min; and holding at 4°C. Amplification products were visualized on a 2% agarose gel and cleaned using a DNA extraction kit (Solarbio, China). The DNA library was prepared with a ligation sequencing kit (SQK-LSK109; Oxford Nanopore Technologies, UK). Briefly, the cleaned DNA was repaired, end repaired, and A-tailed using NEBNext FFPE (formalin-fixed, paraffin-embedded) DNA repair mix (NEB, USA) and the NEBNext Ultra II end repair/Da-tailing module (NEB, USA). Next, the end-repaired samples were ligated with native barcodes using native barcoding expansion 96 (EXP-NBD196; Oxford Nanopore Technologies, UK) and NEB Blunt/TA ligase master mix (NEB, USA). Finally, the barcoded samples were pooled and ligated with adapters using a quick ligation kit (NEB, USA). All samples were purified with Agencourt AMPure XP beads (Beckman, USA). The library was sequenced on a MinION Mk1B platform (Oxford Nanopore Technologies, UK) with an R9.4 flow cell (Oxford Nanopore Technologies, UK).

### Flow cytometry and enzyme-linked immunosorbent assays.

Fresh right lungs and spleens were harvested at 4 days postinfection and dispersed into single-cell suspensions by type 2 collagenase and DNase I (51). Cells were then adjusted to 1 × 10^7^ cells/mL, treated with antibodies, and incubated in the dark for 15 min. After the removal of erythrocytes by using BD Pharm Lyse lysing buffer (BD Biosciences, USA), flow cytometry was performed on a BD FACSCAnto II system (BD Biosciences, USA), and the data were analyzed using FlowJo software (BD Biosciences). Gating strategies are provided in Fig. S1 to S3 in the supplemental material. A list of antibodies used in this study is provided in Table S1. For cellular cytokine analysis, fresh lung tissues collected at 4 days postinfection were homogenized and centrifuged for 5 min at 800 rpm to obtain the supernatants. IL-9 and chemokine (C-C motif) ligand 2 levels were assayed using an Ms inflammation cytometric beads array kit (BioLegend, USA) according to the manufacturer’s instructions. IFN-γ and TNF-α levels were measured using a mouse IFN-γ ELISA kit and a mouse TNF-α ELISA kit (Mreda, China), and IL-8 and IL-6 levels were determined using a mouse IL-8 ELISA kit and a mouse IL-6 ELISA kit (Gersion, China), according to the manufacturers’ instructions.

### Plasma metabolite measurements.

Plasma specimens obtained at 4 days postinfection were prepared using EDTA-2K-anticoagulated fresh blood. A mix of 100 μL of the sample and 300 μL of lysis buffer (80% methanol) was ground for 4 min (40 Hz), sonicated for 5 min (ice water bath), and allowed to stand at −20°C for 30 min. After centrifugation at 14,000 × *g* for 30 min and filtration with a 0.45-μm filter, metabolome profiling was performed using the Thermo Scientific Dionex UltiMate 3000 rapid-separation LC system. For metabolite separation, a Waters Acquity ultraperformance liquid chromatography (UPLC) BEH C_8_ column (1.7 μm, 2.1 mm by 100 mm) was used in electrospray ionization-negative (ESI^−^) mode. Mobile phase A was acetonitrile-water (60/40), and mobile phase B was isopropanol-acetonitrile (90/10); both mobile phases A and B contained 0.1% formic acid and 10 mmol/L ammonium formate. The gradient elution program was performed at 55°C as follows: 98% mobile phase B at 0 min, 98% B at 1 min, 5% B at 6 min, 0% B at 7 min, 0% B at 14.5 min, 98% B at 14.6 min, and 98% B at 16 min. The flow rate was 300 μL/min. Mass spectrometry (MS) was conducted using a Q Exactive hybrid quadrupole Orbitrap mass spectrometer (Thermo Scientific, USA). The spray voltages were set at 3.5 kV, and the heated capillary temperature was 300°C. The parameters for the full mass scan were a resolution of 70,000, an automatic gain control target of <3 × 10^6^, a maximum isolation time of 100 ms, and an *m/z* range of 100 to 1,500. The parameters for the MS/MS scan were a resolution of 17,500, an automatic gain control target of <1 × 10^5^, a maximum isolation time of 50 ms, and normalized collision energies of 10, 30, and 60 V. We then subjected the data to quality control.

### Bioinformatics pipeline.

Raw fast5 files from full-length 16S rRNA gene sequencing were base called using MinKNOW software v3.1.20. Raw reads were then demultiplexed and barcodes were trimmed using the Guppy base caller (v5.0.11+). After filtering out low-quality (average quality of <7) reads, reads with lengths of between 1,200 and 1,800 bp were used for analysis. Clean data were analyzed by using Emu software (52), which uses Minimap (v2.24) and an expectation maximization algorithm to estimate the species composition distribution, using Emu v3.0+ as the reference database. Alpha diversity (Shannon and Chao1 indices) and Bray-Curtis distances were calculated by using the R package vegan (v2.5.7). Principal-coordinate analysis (PCoA) was used to calculate beta diversity by using the R package ape (v5.6.2). PICRUST2 software was used to infer KEGG pathway abundances (53). Heat map and network analyses to correlate the LRT microbiota, immune cells, and metabolites were established by using the R package corrplot and Cytoscape. Plasma metabolite data were collected and processed using Progenesis QI 2.3 software (Waters Corporation, USA) through the Human Metabolome (http://www.hmdb.ca/), METLIN (https://metlin.scripps.edu), and ChemSpider (www.chemspider.com) databases. Differentially expressed metabolites satisfied the following conditions: an average fold change ratio of >2, a *P* value of <0.05, and a variable importance in projection (VIP) value of >1. Pathway analysis was performed using KEGG pathways (KEGG protein database [http://www.kegg.jp/kegg/pathway.html]). Random-forest analysis was performed as described previously (54).

### Statistics.

Body weights, pathogen loads, and levels of immune cells, metabolites, and cytokines were compared by one-way analysis of variance (ANOVA) for multiple-group analysis. Shannon and Chao1 indices and bacterial taxon abundances among groups were compared by using the Kruskal-Wallis test. The correlations among strains, immune cells, and metabolites were determined by employing the R package Hmisc rcorr using the Spearman test. A *P* value of <0.05 was considered statistically significant.

### Ethics approval and consent to participate.

All animal procedures for animal raising and handling were approved by the Animal Care and Use Committee of the Chinese PLA Center for Disease Control and Prevention.

### Data availability.

Full-length 16S rRNA gene sequencing data were deposited in the NCBI database (https://www.ncbi.nlm.nih.gov/bioproject/) under BioProject accession number PRJNA900717. The assembled methicillin-resistant Staphylococcus aureus strain BJFAN203 genome was deposited in the GenBank database under accession number JAPKAA000000000. The data and code required to reproduce all analyses are available from https://github.com/PCDClilab/mouse_respirator_tract_microbiome.
