# Supplementary material for: Altered Respiratory Microbiomes, Plasma Metabolites, and Immune Responses in Influenza A Virus and Methicillin-Resistant Staphylococcus aureus Coinfection
Source: Microbiol Spectr. 2023 Jun 15;11(4):e05247-22. doi: 10.1128/spectrum.05247-22 (PMC10433956; doi:10.1128/spectrum.05247-22)
Supplement: Supplemental file 1 — Supplemental material. Download spectrum.05247-22-s0001.pdf, PDF file, 1.4 MB [file spectrum.05247-22-s0001.pdf]

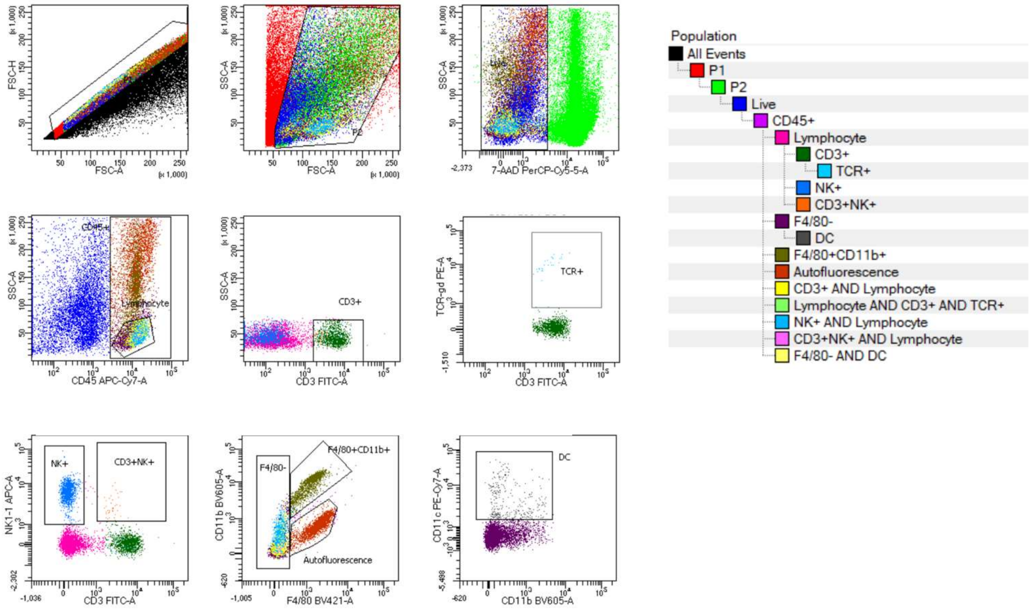

Supplementary Fig. S1: Gating strategy for identification of macrophage, DC, NK and γδT cell by flowcytometry.

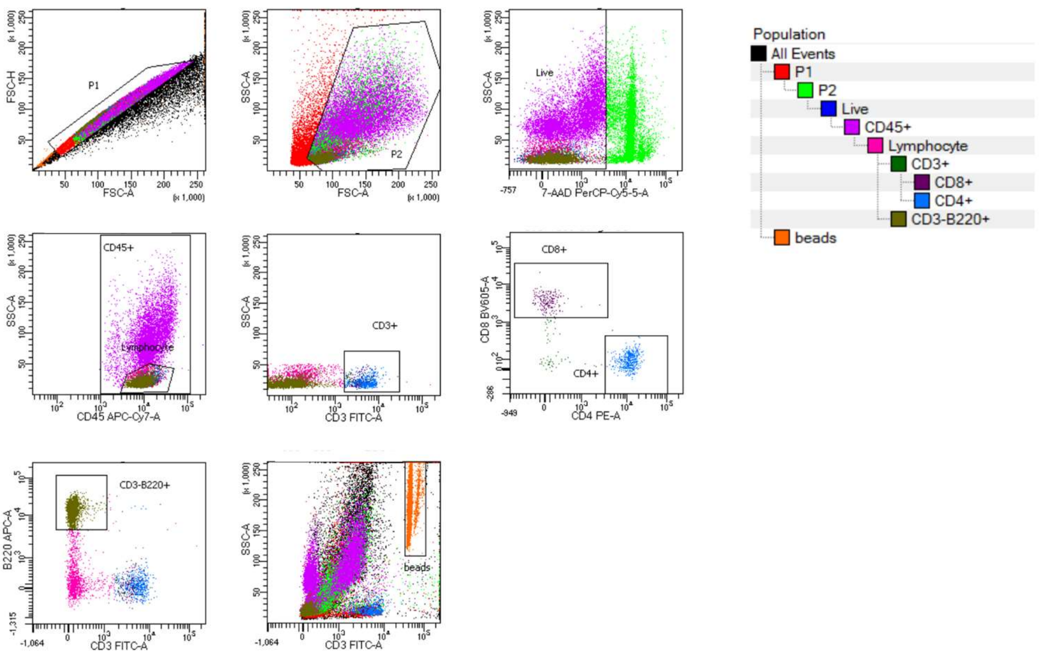

Supplementary Fig. S2: Gating strategy for identification of CD4+, CD8+ T cell and B cell by flowcytometry.

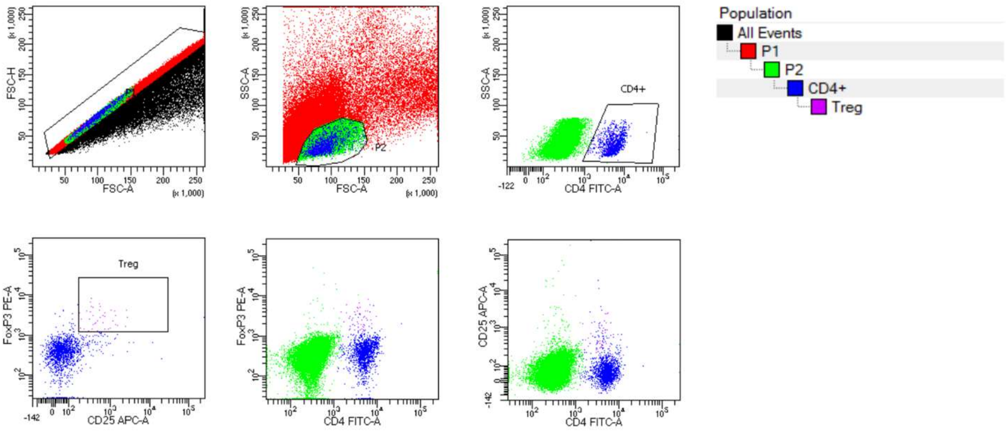

Supplementary Fig. S3: Gating strategy for identification of Treg cell by flowcytometry.

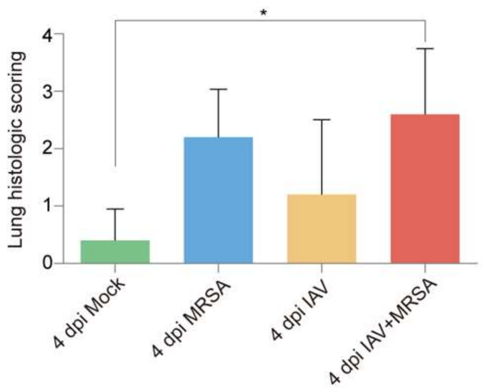

Supplementary Fig. S4: Histologic scoring of lung at 4 days post-infection for each group. Statistics for histologic scoring were analyzed by the Kruskal-Wallis test, * $P<0.05$.

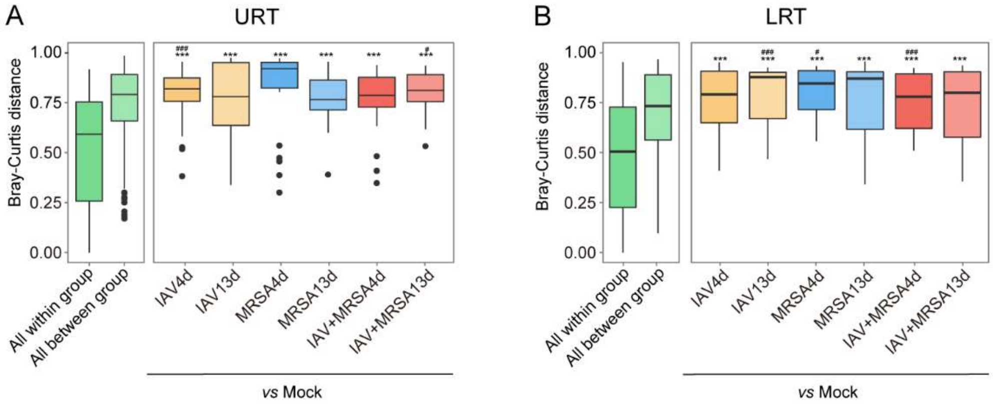

Supplementary Fig. S5: Bray-Curtis distances of LRT and URT microbiota at 4 and 13 days post-infection for each group. (A) Bray-Curtis distance of URT microbiota for each group. (B) Bray-Curtis distance of LRT microbiota each group. URT, upper respiratory tract; LRT, lower respiratory tract; dpi, days post-infection. Statistics for Bray-Curtis distances were analyzed by the Kruskal-Wallis test, * towards all-within group, # towards all between group.

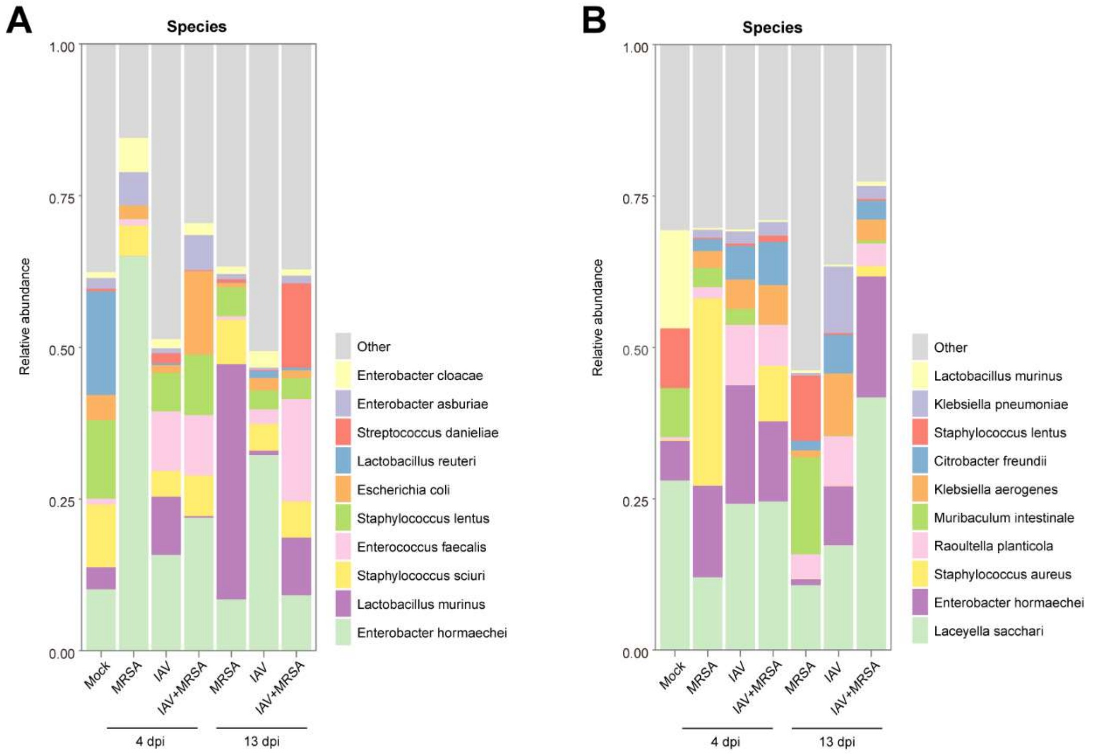

Supplementary Fig. S6: URT and LRT microbiomes at species level for all groups at 4 and 13 days post-infection. (A) Relative abundance of top 10 URT microbiota at the species level in all groups. (D) Relative abundance of top 10 LRT microbiota at the species level in all groups. URT, upper respiratory tract; LRT, lower respiratory tract; dpi, days post-infection.

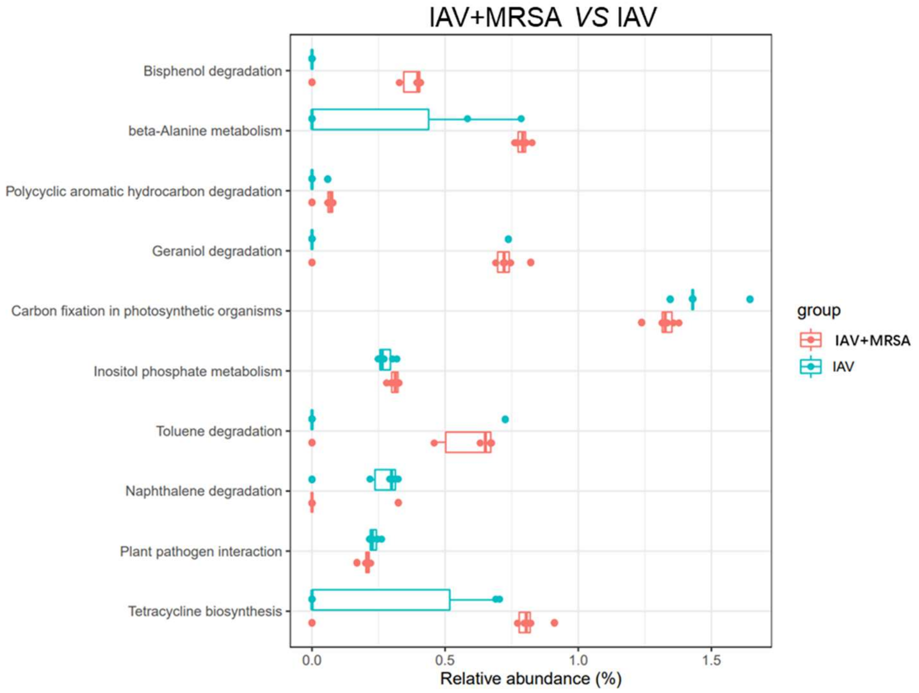

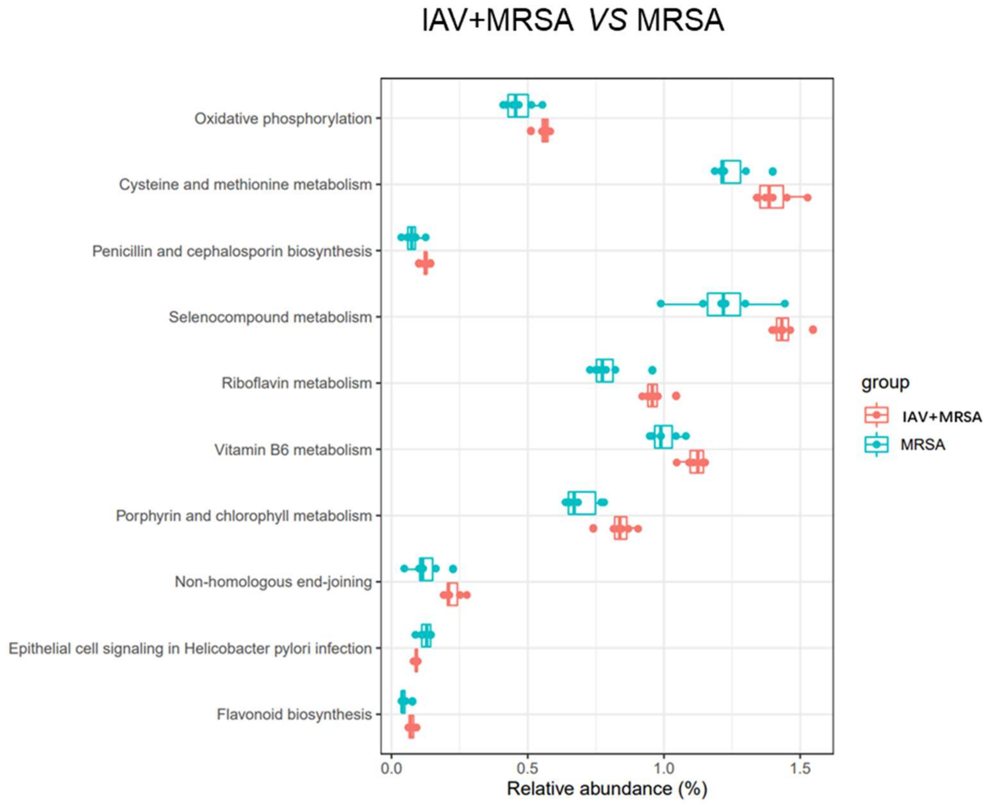

Supplementary Fig. S7: Predicted KEGG metabolism pathways of LRT microbiota at 4 days post-infection

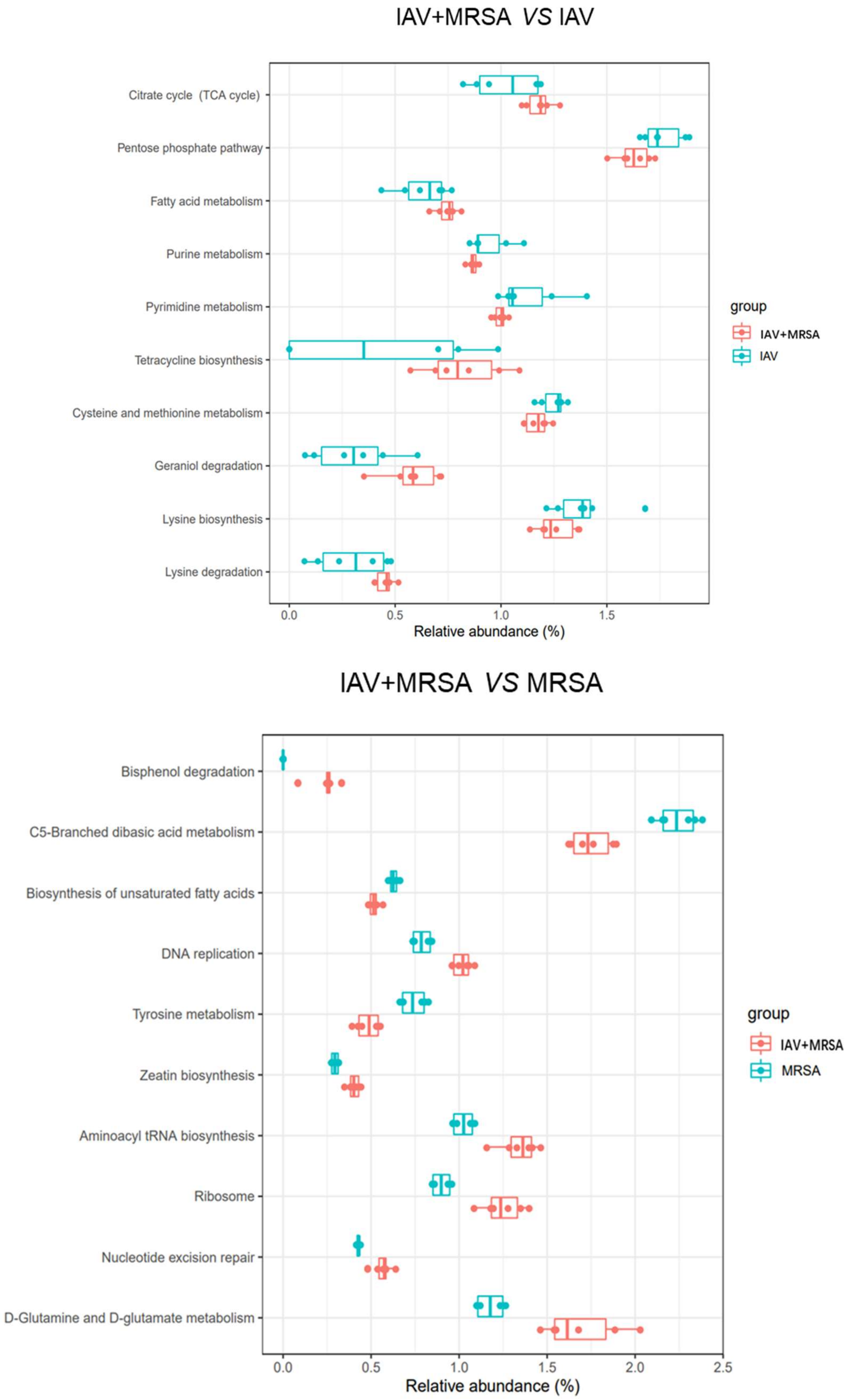

Supplementary Fig. S8: Predicted KEGG metabolism pathways of LRT microbiota at 4 days post-infection.

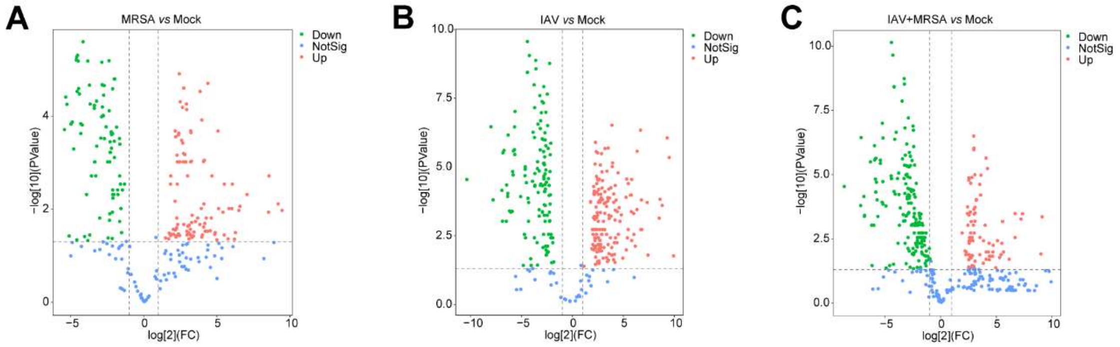

Supplementary Fig. S9: Significantly differential metabolites between infected and mock-infected groups at 4 days post-infection. (A) Differentiating metabolites between MRSA group and Mock group. (B) Differentiating metabolites between IAV group and Mock group. (C) Differentiating metabolites between IAV+MRSA group and Mock group. Metabolite levels were compared with one-way ANOVA for multiple groups analysis. A *P*-value of < 0.05 was considered statistically significant.

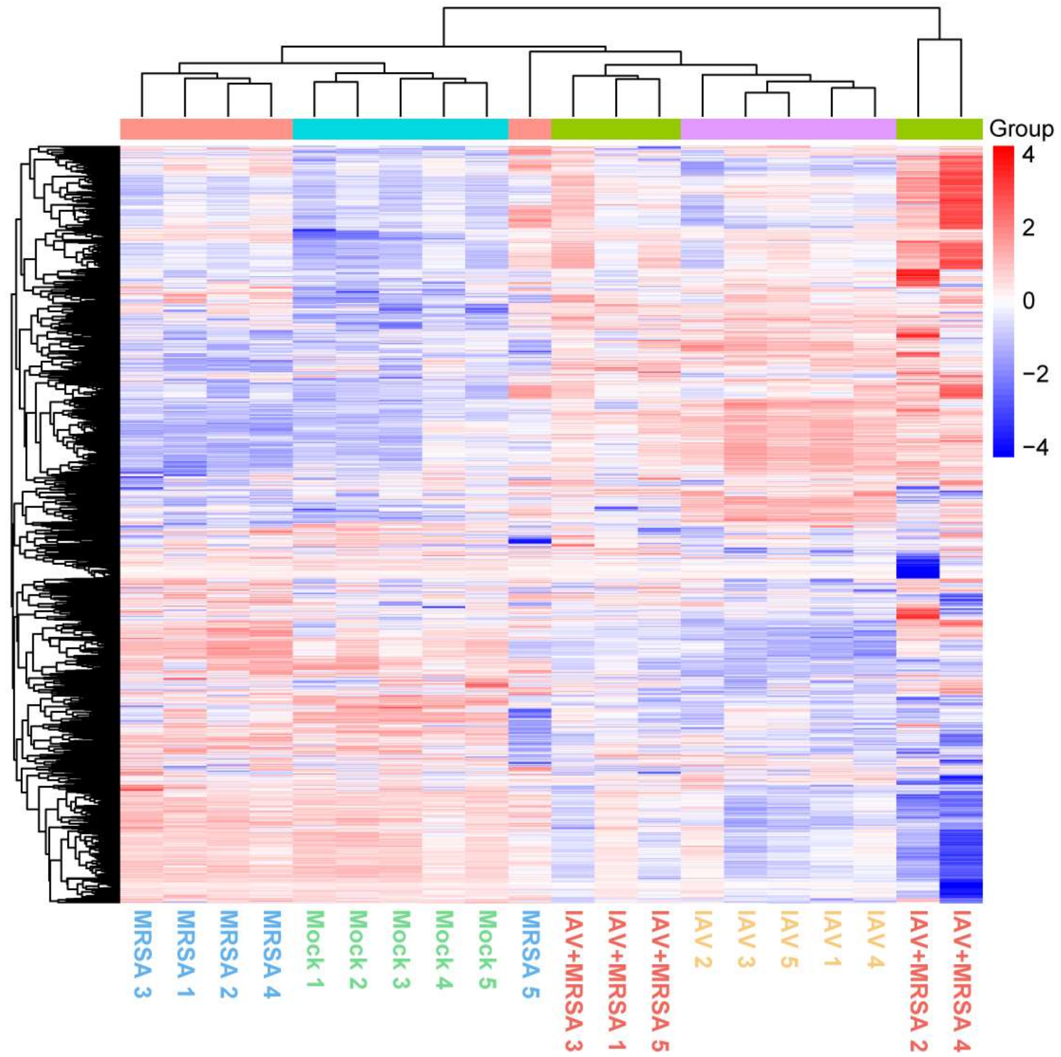

Supplementary Fig. S10: Heatmap and clustering analyses of plasma metabolites of all samples at 4 days post-infection.

Supplementary Table. S1: List of antibodies used in this study

ANTIBODIES	SOURCE	CATALOG #
CD3-FITC	Biolegend	100204
TCR-γσ-PE	Biolegend	118108
CD11b-BV605	Biolegend	101237
NK1.1-APC	BD Pharmingen	550627
CD11c-PE/Cy7	BD Pharmingen	558079
CD45-APC/Cy7	Biolegend	103116
F4/80-BV421	Biolegend	123132
7-AAD	Biolegend	420404
CD4-PE/Cy7	Biolegend	10042
CD8-BV605	Biolegend	563152
B220-APC	Biolegend	103211
CD4-FITC	BD Pharmingen	553046
CD25-APC	BD Pharmingen	557192
FoxP3-PE	Invitrogen	12-5773-82

Supplementary Table. S2: Significantly different species in the IAV+MRSA group versus the IAV or MRSA group

Species	Site	Time	Group1	Group	adjusted.pvalue
Streptococcus danieliae	URT	4dpi	IAV+MRSA	MRSA	0.028865159
Staphylococcus lentus	URT	4dpi	IAV+MRSA	MRSA	0.014994374
Staphylococcus lentus	LRT	4dpi	IAV+MRSA	MRSA	0.006493506
Muribaculum intestinale	LRT	13dpi	IAV+MRSA	MRSA	0.024871834
Lactobacillus murinus	URT	4dpi	IAV+MRSA	MRSA	0.02338748
Laceyella sacchari	LRT	13dpi	IAV+MRSA	MRSA	0.045454545
Enterococcus faecalis	URT	4dpi	IAV+MRSA	MRSA	0.006493506
Enterococcus faecalis	URT	13dpi	IAV+MRSA	MRSA	0.006493506
Enterobacter hormaechei	URT	4dpi	IAV+MRSA	MRSA	0.006493506
Enterobacter hormaechei	LRT	13dpi	IAV+MRSA	MRSA	0.017316017
Enterobacter cloacae	URT	4dpi	IAV+MRSA	MRSA	0.006493506
Staphylococcus aureus	LRT	4dpi	IAV+MRSA	IAV	0.00833529
Klebsiella pneumoniae	LRT	13dpi	IAV+MRSA	IAV	0.024871834
Enterococcus faecalis	URT	13dpi	IAV+MRSA	IAV	0.008658009
Enterobacter hormaechei	URT	13dpi	IAV+MRSA	IAV	0.006493506
Enterobacter cloacae	URT	13dpi	IAV+MRSA	IAV	0.006493506

URT, upper respiratory tract; LRT, lower respiratory tract; dpi, days post-infection.
